# Image-based robotic-assisted patellofemoral arthroplasty for isolated patellofemoral osteoarthritis: Short-term survivorship, clinical and radiographic outcomes

**DOI:** 10.1051/sicotj/2026070

**Published:** 2026-10-12

**Authors:** Alberto Fogacci, Clément Favroul, Luca Andriollo, Pierre Longlune, Cécile Batailler, Elvire Servien, Sébastien Lustig

**Affiliations:** 1 Orthopaedics Surgery and Sports Medicine Department, FIFA Medical Center of Excellence, Croix-Rousse Hospital, Hospices Civils de Lyon, Lyon North University Hospital 103 Grande Rue de la Croix-Rousse 69004 Lyon France; 2 IRCCS Istituto Ortopedico Rizzoli, II Orthopaedic Clinic Bologna Italy; 3 Orthopaedics and Traumatology, Fondazione Poliambulanza Istituto Ospedaliero Brescia Italy; 4 Univ Lyon, Claude Bernard Lyon 1 University IFSTTAR, LBMC UMR_T9406 69622 Lyon France; 5 LIBM-EA 7424, Interuniversity Laboratory of Biology of Mobility, Claude Bernard Lyon 1 University Lyon France

**Keywords:** Patellofemoral arthroplasty, Robotic-assisted surgery, Survivorship

## Abstract

*Introduction*: Patellofemoral arthroplasty (PFA) is a valuable option for selected patients with isolated patellofemoral osteoarthritis (PFOA), where component positioning and patellar tracking are critical determinants of outcome. Robotic assistance may improve these aspects, but available literature remains limited. This study evaluated early survivorship, complications, and reoperations after image-based robotic-assisted PFA (RA-PFA). Secondary aims were to assess clinical outcomes, return to sport, radiographic patellofemoral parameters, and agreement between planned and final implanted component position. *Method:* This retrospective single-center study included consecutive patients treated with RA-PFA for isolated PFOA between November 2021 and October 2024, with a minimum clinical and radiographic follow-up of 12 months. Primary outcomes were implant survivorship, reoperation-free survivorship, and postoperative complications. Secondary outcomes included Knee Society Score (KSS), range of motion (ROM), return to sport, radiographic patellofemoral parameters, and MAKO-derived plan-to-execution agreement. Kaplan–Meier analysis was used to assess survival. *Results*: Thirty patients (35 knees; 5 bilateral cases) were included, with a mean age of 61.3 ± 12.2 years and mean body mass index of 25.3 ± 5.2 kg/m^2^. Most knees had advanced disease (Iwano grade III–IV, 91.4%). Mean follow-up was 15.7 ± 8.5 months. Overall reoperation rate was 8.6% (3/35), including two conversions to TKA. Survivorship free from revision was 96.7% (95% CI 90.2–100.0) at 12 months and 91.6% (95% CI 80.1–100.0) at 24 and 36 months. KSS improved significantly (from 110.7 ± 21.1 to 163.7 ± 22.3; *p* < 0.001). Median ROM remained 130°. Return to sport was achieved in 78.3% of active patients. Patellar lateralization improved significantly (4.7 ± 3.8 mm to 0.8 ± 1.8 mm; *p* < 0.001), with a reduction in patellar tilt. Plan-to-execution deviations were minimal, with 100% component size concordance. *Discussion:* Image-based RA-PFA was associated with encouraging early survivorship, substantial functional gains, preserved knee motion, and improved radiographic patellofemoral alignment at short-term follow-up. However, the specific contribution of robotic assistance cannot be determined in the absence of a conventional control group. *Level of evidence*: Level IV.

## Introduction

Isolated patellofemoral osteoarthritis (PFOA) is a relevant cause of anterior knee pain and functional limitation, frequently associated with trochlear dysplasia and abnormal patellar tracking. Epidemiological data suggest that patellofemoral involvement represents a substantial proportion of compartmental knee osteoarthritis, supporting the clinical relevance of isolated patellofemoral disease [1, 2]. Symptoms are often disproportionate to the extent of tibiofemoral degeneration and are typically exacerbated by activities like stair climbing, squatting, and prolonged sitting, supporting the need for compartment-specific treatment strategies in appropriately selected patients. Patellofemoral arthroplasty (PFA) has regained interest with modern implant designs and refined indications [3]. Current evidence suggests that favorable results depend on meticulous patient selection, including the presence of isolated advanced symptomatic PFOA, absence of clinically relevant tibiofemoral disease, acceptable limb alignment, and restoration of satisfactory patellar tracking [4–6]. In modern series, failure is more commonly related to disease progression and technical factors rather than implant design itself [5, 6].

Accurate component positioning is particularly critical in PFA, as implant size, rotation, sagittal alignment, and anterior offset directly influence patellofemoral mechanics and clinical outcomes. [5, 6] CT-based robotic assistance enables patient-specific three-dimensional planning with intraoperative refinement, potentially improving reproducibility of implant positioning and allowing patellar tracking assessment in anatomically complex joints [4–7].

Early reports on robotic-assisted PFA (RA-PFA) have shown promising short-term survivorship and outcomes [5, 8–10]. However, available evidence remains limited by small series, short follow-up, and incomplete reporting of radiographic tracking parameters and plan-to-execution agreement. In particular, the relationship between planned component position, intraoperative execution, and postoperative patellofemoral tracking has not been fully characterized. The primary aim of this study was to evaluate early implant survivorship, reoperations, and complications after image-based RA-PFA. Secondary aims were to assess clinical outcomes, return to sport, radiographic patellofemoral parameters, and agreement between preoperative planning and final implanted component position.

## Materials and methods

### Study design

This retrospective single-center cohort study included a consecutive series of patients who underwent CT-based RA-PFA for isolated PFOA between November 2021 and October 2024. Patients with a minimum clinical and radiographic follow-up of 12 months were included in the analysis.

Primary outcomes were implant and reoperation-free survivorship and postoperative complications. Implant survivorship was defined as freedom from revision of any prosthetic component for any reason. Reoperation-free survivorship was defined as freedom from any secondary surgical procedure on the index knee. Secondary outcomes were clinical and functional improvement, return to sport, radiographic patellofemoral parameters, and agreement between planned and final implanted trochlear component position.

### Study population

Eligible patients were adults aged 18 years or older who underwent isolated RA-PFA for symptomatic isolated PFOA. Indications were based on the presence of isolated symptomatic patellofemoral disease considered suitable for arthroplasty after failure of non-operative treatment [6].

Patients were excluded if they had clinically or radiographically relevant tibiofemoral OA, underwent bicompartmental or tricompartmental arthroplasty at index procedure, or had severe systemic conditions considered likely to materially affect postoperative functional outcomes. Baseline demographic and clinical characteristics of the final cohort are reported in the Results section (Table 1).

**Table 1:** Demographic and baseline clinical characteristics of the study population.

Variable	Overall cohort (*n* = 30 patients, 35 knees)
Age at surgery (years) mean ± SD	61.3 ± 12.2
BMI (kg/m^2^) mean ± SD	25.3 ± 5.2
Gender distribution n ( %)	
Male	6 (20%)
Female	24 (80%)
Side *n* (%)	
Left	11 (36.7%)
Right	14 (46.7%)
Bilateral	5 (16.7%)
Prior surgery on the index knee, *n* (%)	
Yes	11 (31.4%)
No	24 (68.6%)
Number of previous surgeries index knee, *n* (%)	
0	24 (68.6%)
1	9 (25.7%)
>1	2 (5.7%)
Type of previous surgery index knee, n	
TTO	4
TTO + VMO plasty	1
Lateral release and medial reinforcement	1
Lateral hemipatellectomy	2
UKA	3
High tibial osteotomy	2
Patella fracture fixation	1
Osteoarthritis stage (IWANO classification), *n* (%)	
1	0
2	3 (8.6%)
3	17 (48.6%)
4	15 (42.8%)
Anesthesia, *n* (%)	
General	6 (20%)
Spinal	24 (80%)
Surgical time (minutes) mean ± SD	58.8 ± 17.9
PFA femur size, *n* (%)	
2	14 (40%)
3	12 (34.3%)
4	4 (11.4%)
5	2 (5.7%)
6	3 (8.6%)
PFA patella size (mm), *n* (%)	
26	1 (2.9%)
29	5 (14.3%)
31	4 (11.4%)
32	11 (31.4%)
33	2 (5.7%)
35	9 (25.7%)
38	3 (8.6%)
Follow-up (months) mean ± SD	15.7 ± 8.5

### Surgical technique

All procedures were performed by three high-volume knee surgeons (SL, ES, CB), using a midvastus surgical approach.

Restoris^®^ MCK patellofemoral system was implanted utilizing the MAKO Robotic Arm Interactive Orthopedic System based on a preoperative CT scan (Stryker Corp, Mako Surgical Corp, Ft. Lauderdale, FL, USA).

CT scan was used to define implant size and three-dimensional femoral component positioning (coronal alignment, axial rotation, sagittal flexion, and proudness). Intraoperatively, diaphyseal bicortical femoral arrays were fixed, bony landmarks registered, and trochlear mapping performed. CT-based plan was then reviewed and refined in real time according to patient-specific anatomy and native patellar tracking recorded, with adjustment of component size and position when required before robotic bone preparation. After intraoperative adjustment, robotic arm-assisted trochlear resection was performed with a burr, and trial components were inserted. Patellar resection was performed with conventional instrumentation. Final implantation aimed to restore anterior offset and balanced patellar tracking before definitive cementation. [4, 11] (Figures 1, 2).

**Figure 1:**
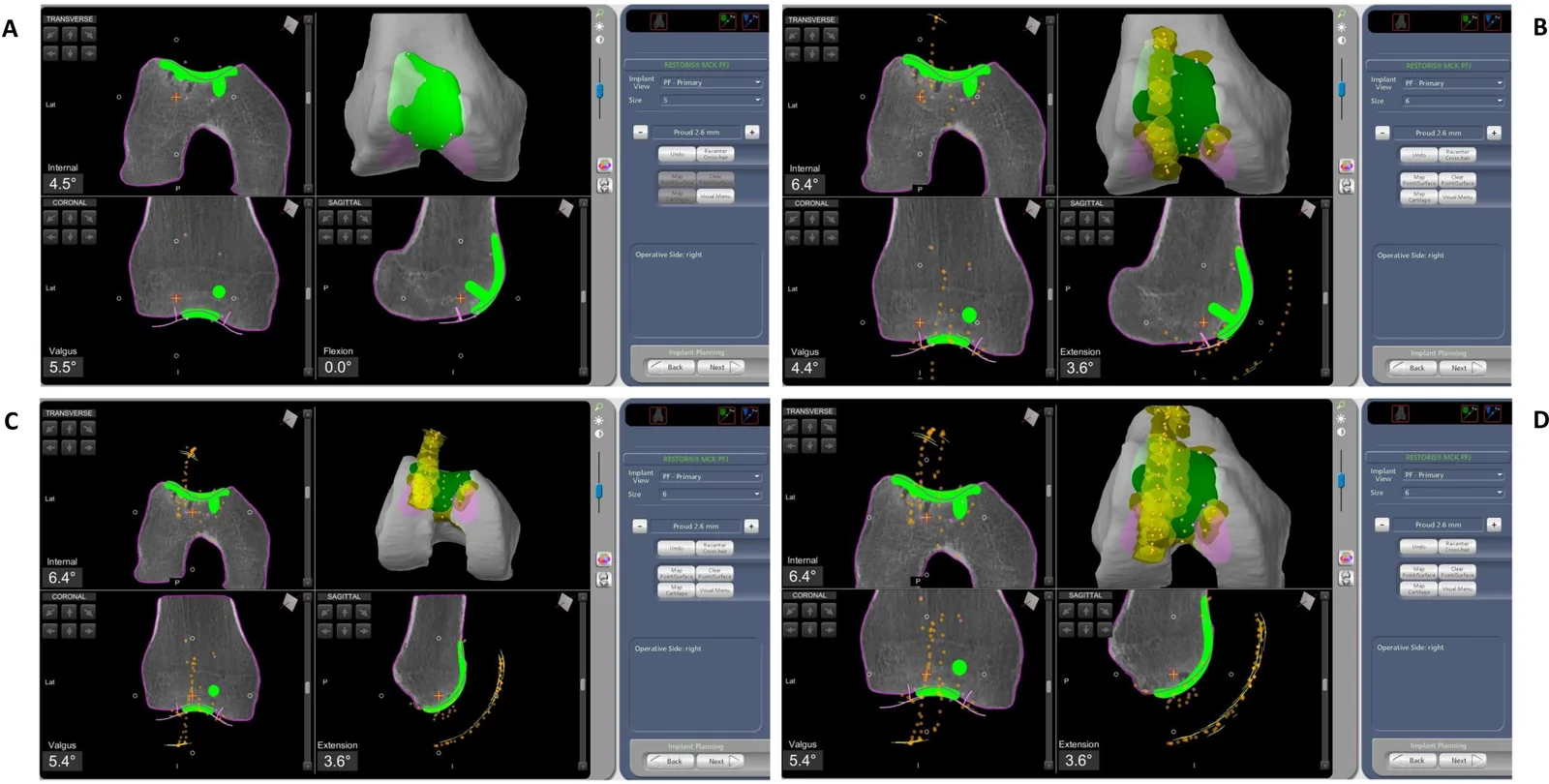
Representative CT-based preoperative planning and intraoperative workflow of MAKO RA-PFA (A) Initial preoperative planning. (B) Intraoperative registration of anatomic landmarks, cartilage surface, and native patellar tracking. (C) Adjustment of the preoperative plan to the patient’s native anatomy based on intraoperative registration. (D) Final implant positioning with final assessment of patellar tracking.

**Figure 2:**
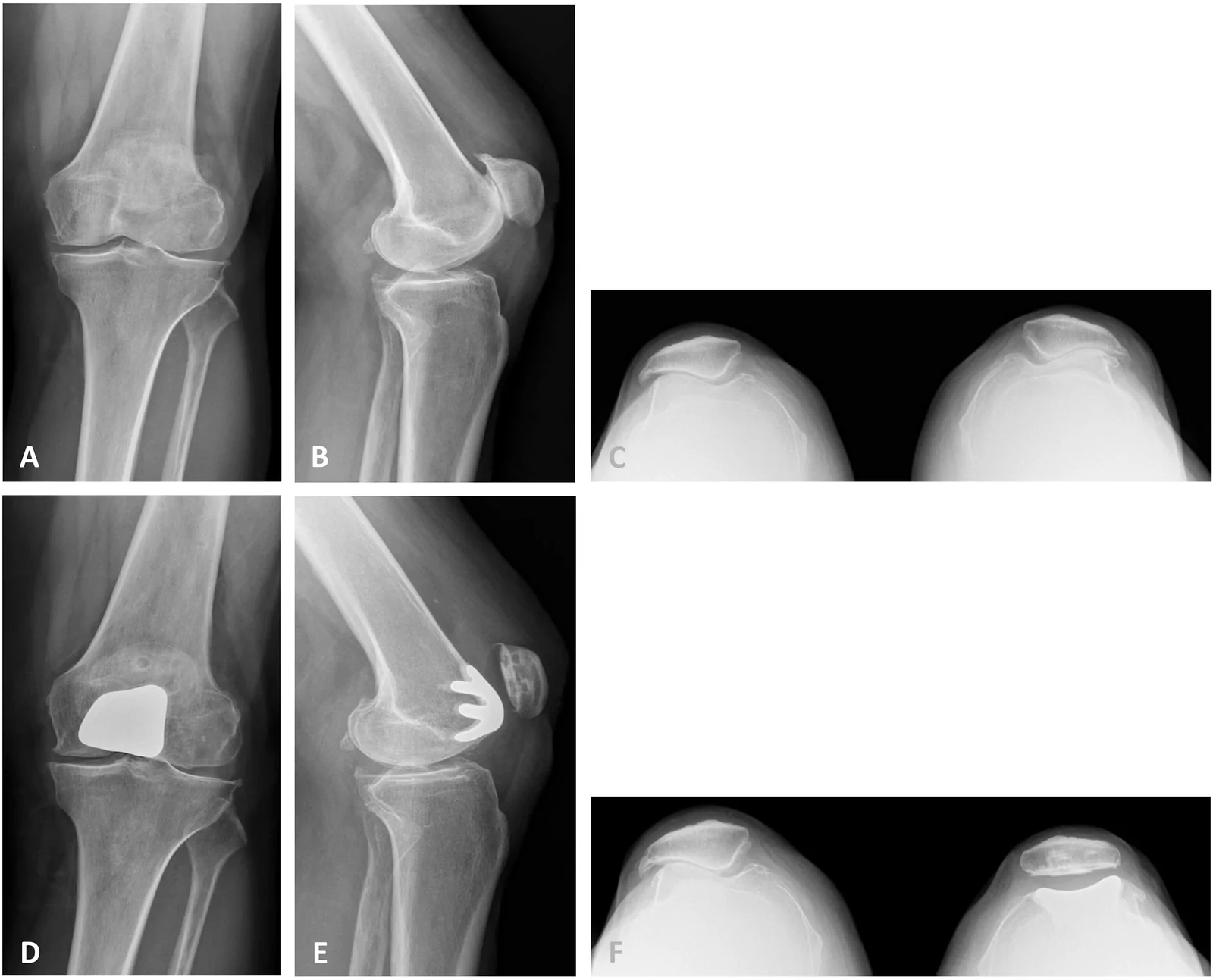
Representative clinical case illustrating pre (A) and postoperative (B) imaging after robot-assisted patellofemoral arthroplasty

### Data assessment

All follow-up data were collected at scheduled outpatient control, including clinical and radiographic evaluation. Demographic data were obtained from medical records.

Weight-bearing anteroposterior and lateral knee radiographs, patellar axial views, and full-length standing radiographs were performed preoperatively and at the time of follow-up. PFOA severity was assessed according to Iwano classification, while tibiofemoral osteoarthritis was graded according to the Kellgren–Lawrence (KL) classification.

Radiographic parameters included hip-knee-ankle (HKA) angle, Caton-Deschamps Index, patellar tilt, sulcus angle, patellar lateralization, congruence angle, and anterior trochlear offset ratio (ATOR). Patellar tilt, congruence angle, and patellar lateralization were measured on standardized patellar skyline (Merchant) radiographs obtained at 45° of knee flexion, according to the methods illustrated in Figure 3. Patellar lateralization was recorded as a directional measurement, with positive values indicating lateral displacement and negative values indicating medial displacement. ATOR was measured on a lateral radiograph as the ratio between the anteroposterior width of the trochlear component and the femoral shaft [12].

**Figure 3:**
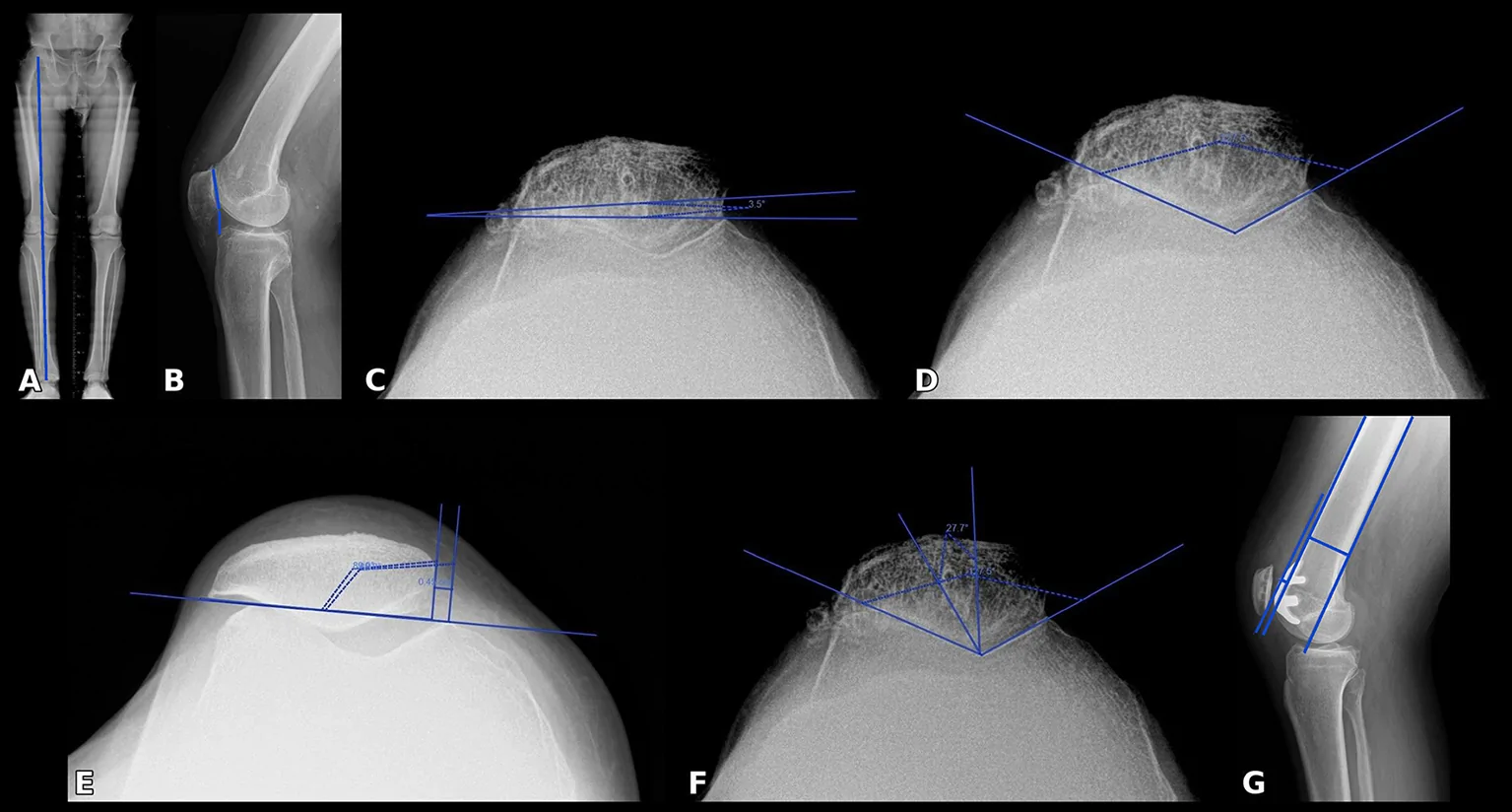
Radiographic parameters assessed. (A) HKA; (B) Caton-Deschamps index; (C) patellar tilt; (D) sulcus angle; (E) patellar lateralization; (F) congruence angle; (G) ATOR

All measurements were performed by independent reviewers using predefined sign conventions (positive values indicating lateral deviation for patellar parameters). Formal inter- and intra-rater reliability analyses were not performed. However, the radiographic parameters used in this study were measured according to standardized methods and have previously shown good to excellent reproducibility in the literature. Representative measurement methods are shown in Figure 3.

Clinical evaluation included the Knee Society Score (KSS) and range of motion (ROM) assessed preoperatively and at final follow-up. Preoperative sports participation was recorded as part of the patient’s medical history. At final follow-up, return to sport (RTS) was assessed by patient report and classified according to activity impact (low-, medium-, or high-impact), while activity level was quantified using the Tegner Activity Scale. No predefined criteria regarding frequency, intensity, time to return, or return to the same performance level were applied. All complications and reoperations were documented. Preoperative planning and intraoperative data were extracted from the MAKO system database, including component position in coronal, sagittal, and axial planes, as well as proudness. Sign convention was predefined as follows: varus, flexion, external rotation, and over-bone proudness were considered positive. Plan-to-execution values represented intraoperative component positioning recorded by the MAKO system and were not independently validated using postoperative CT imaging.

### Statistical analysis

Continuous variables are presented as mean ± standard deviation or median with interquartile range, according to distribution. Categorical variables are presented as counts and percentages. Preoperative-to-postoperative changes were assessed using paired *t*-tests or Wilcoxon signed-rank tests, depending on normality testing with the Shapiro–Wilk test.

Agreement between planned and final implanted component position was assessed by testing the difference between planned and intraoperative values against zero. Survivorship was estimated using Kaplan–Meier analysis with two endpoints: revision of any prosthetic component and any reoperation. Patients were censored at last follow-up. A *p*-value <0.05 was considered statistically significant.

Because only five patients underwent bilateral procedures, knees were analyzed as independent observations, and no adjustment for within-patient correlation was performed.

## Results

### Baseline characteristics

30 patients (35 knees, including 5 bilateral cases) were included: 24 women, 6 men. Mean age at surgery was 61.3 ± 12.2 years and mean BMI was 25.3 ± 5.2 kg/m^2^.

At baseline, according to the Iwano classification, PFOA was stage 2 in 3 knees (8.6%), stage 3 in 17 knees (48.6%), and stage 4 in 15 knees (42.8%), with the majority presenting with advanced patellofemoral disease (stage 3–4: 91.4%). All knees demonstrated absent or mild tibiofemoral osteoarthritis, with 12 knees (34.3%) classified as KL grade 0 and 23 knees (65.7%) as KL grade 1; no knee demonstrated moderate or advanced tibiofemoral osteoarthritis (KL grades 2–4). Previous surgery on the index knee had been performed in 11/35 knees (31.4%), including one prior procedure in 9 knees (25.7%) and more than one in 2 knees (5.7%). Patellar realignment procedures for maltracking or instability were reported in 4/35 knees (11.4%).

Mean follow-up was 15.7 ± 8.5 months (Table 1).

### Complications, reoperations and survivorship

Postoperative adverse events and pain-related symptoms occurred in 9/35 knees (25.7%). (Table 2) Persistent postoperative pain was defined as pain arising or worsening after surgery, localized to the operated knee/peripatellar region, lasting >6 months, and not attributable to infection, trauma, implant-related failure, or other identifiable causes after routine clinical and radiographic evaluation. Pain-related symptoms were observed in 6 knees: 4 cases of isolated persistent pain, 1 case associated with medial dysesthesia and algodystrophy, and 1 case of worsened pain compared to baseline with quadriceps atrophy. Other adverse events included quadriceps tendon tendinopathy (*n* = 1), patellar instability (*n* = 1), and 10° flexion contracture (*n* = 1). Most non-reoperated adverse events were managed conservatively and showed symptom improvement at final follow-up.

**Table 2:** Postoperative complications, reoperations, and implant failures.

Case	Complication category	Main event	Associated findings	Note	Reoperation	Treatment and outcomes
Case 1	Pain-related symptom	Persistent postoperative pain	/	Over 1 year post op	No	Conservative management; symptom improvement at final follow-up.
Case 2	Soft-tissue complication	Quadriceps tendon tendinopathy	/	/	No	Conservative management; symptom improved without surgery.
Case 3	Mechanical complication	Patellar instability	/	/	Yes	TTO + MPFL reconstruction
Case 4	Stiffness	Flexion contracture	/	ROM 10–105°	No	Conservative management; symptom improved without surgery.
Case 5	Pain-related symptom	Persistent postoperative pain	/	Over 1 year post op	Yes	Conversion to TKA
Case 6	Pain-related symptom	Persistent postoperative pain	Quadriceps atrophy	Over 6 months post op, worse than preoperative	No	Conservative management; persistent symptom at final follow-up.
Case 7	Pain-related symptom	Persistent postoperative pain	/	Over 19 months post op	No	Conservative management; symptom improvement at final follow-up.
Case 8	OA progression	Progressive tibiofemoral OA	/	Progression tibio femoral OA	Yes	Conversion to TKA
Case 9	Pain-related symptom	CRPS/algodystrophy	Algodystrophy	Over 1 year post op	No	Conservative management; symptom improvement at final follow-up.

Reoperation was required in 3/35 knees (8.6%): two revisions to TKA at 9 and 13 months (one for persistent pain with tibiofemoral OA progression, one for persistent pain), and one TTO distalization with MPFL reconstruction for patellar instability at 12 months in a patient without prior instability surgery.

At the latest follow-up, crude survivorship free from revision of any prosthetic component was 94.3% (33/35 knees), while crude survivorship free from any reoperation was 91.4% (32/35 knees). Kaplan–Meier estimated revision-free survivorship was 96.7% (95% CI, 90.2–100.0) at 12 months and 91.6% (95% CI, 80.1–100.0) at both 24 and 36 months. Kaplan–Meier estimated reoperation-free survivorship was 92.1% (95% CI, 81.3–100.0) at 12 months and 86.9% (95% CI, 72.9–100.0) at both 24 and 36 months.

Because only a limited number of knees remained at risk beyond 24 months, the estimates at 24 and 36 months should be interpreted cautiously (Figure 4).

**Figure 4:**
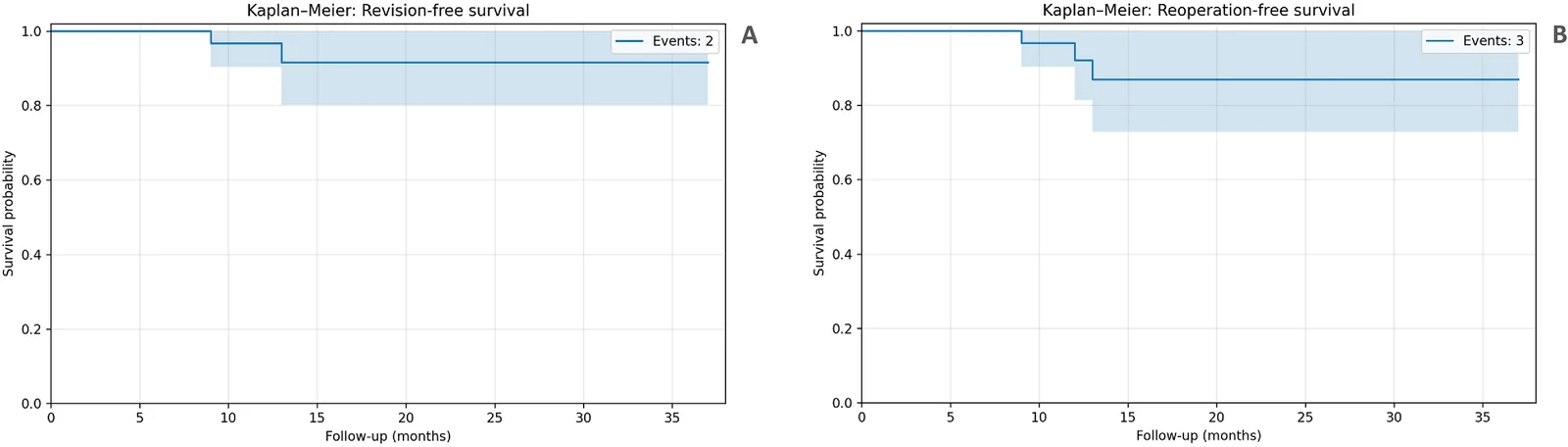
(A) Kaplan–Meier: revision of prosthetic component for any reason. (B) Kaplan–Meier: reoperation-free survival. Numbers at risk are reported below the curves. Revision-free survival: 0 months: 35; 12 months: 34; 24 months: 9; 36 months: 1. Reoperation-free survival: 0 months: 35; 12 months: 34; 24 months: 9; 36 months: 1.

### Plan-to-execution agreement

Mean difference between intraoperative implant position and preoperative planning was −0.19 ± 0.75 degrees for flexion/extension (*p* = 0.2201), 0.23 ± 1.19 degrees for varus/valgus (*p* = 0.29), 0.12 ± 0.45 degrees for external/internal rotation (*p* = 0.25), −0.12 ± 0.46 mm for proudness (*p* = 0.16).

Planned and implanted trochlear component sizes were fully concordant.

No significant differences were found between planned and final component positioning across sagittal, coronal, axial alignment, and proudness (all *p* > 0.05), indicating absence of systematic deviation from preoperative plan (Table 3).

**Table 3:** Radiographic outcomes and MAKO-derived plan-to-execution parameters.

Variable	Pre-operative (Mean ± SD)	Post-operative (Mean ± SD)	Δ Post-op − pre-op (Mean ± SD)	Δ |Post-op − pre-op| (Mean ± SD)	*p*-value
Sagittal flexion /extension (°)	4.58 ± 3.37	4.45 ± 3.24	−0.19 ± 0.75	0.55 ± 0.56	*p* = 0.2201
Coronal varus/valgus (°)	0.58 ± 2.39	0.82 ± 2.95	0.23 ± 1.19	0.86 ± 0.84	*p* = 0.2987
Axial ext rotation/intern rotation (°)	0.60 ± 3.95	0.71 ± 4.06	0.12 ± 0.45	0.39 ± 0.28	*p* = 0.2477
Proudness pre-op (mm)	1.14 ± 0.80	1.02 ± 0.81	−0.12 ± 0.46	0.39 ± 0.26	*p* = 0.1609
Flexion (+) / Extension (−); Varus (+) / Valgus (−); External rotation (+) / Internal rotation (−); Proudness/Over (+) / Under (−)					
HKA (°)	179.6 ± 2.8	/	/	/	/
**Trochlear** sulcus angle (°)	137.2 ± 8.3	/	/	/	/
Congruence angle (°)	28.4 ± 23.9	/	/	/	/
Patellar tilt (°)	8.8 ± 6.7	6.4+4.2	−2.4+9.3	6.9+6.1	*p* = 0.139
Caton-Deschamps index	1.1 ± 0.3	1.0+0.1	−0.08+0.32	/	*p* = 0.225
Patellar lateralization (mm)	4.7 ± 3.8	0.8 ± 1.8	−3.9 ± 3.5	4.2 ± 3.1	*p* = <0.0001
ATOR index	/	0.12 ± 0.05			/
Sign convention: Patellar Tilt, Congruence Angle, Patellar Lateralization, Lat* Med −					

Radiographic parameters are reported as pre- and postoperative values, while MAKO parameters represent the deviation between planned and intraoperative component position.

### Clinical and functional evaluation

Clinical and functional outcomes, assessed with KSS, improved significantly across all domains (all *p* < 0.001): clinical from 64.6 ± 11.8 to 83.6 ± 12.1 corresponding to a mean improvement of 19.0 points (95% CI, 13.9–24.1), functional from 46.1 ± 17.2 to 80.1 ± 14.0 with a mean increase of 34.0 points (95% CI, 26.5–41.5), the global from 110.7 ± 21.1 to 163.7 ± 22.3 representing a mean improvement of 53.0 points (95% CI, 43.0–63.0) (Figure 5).

**Figure 5:**
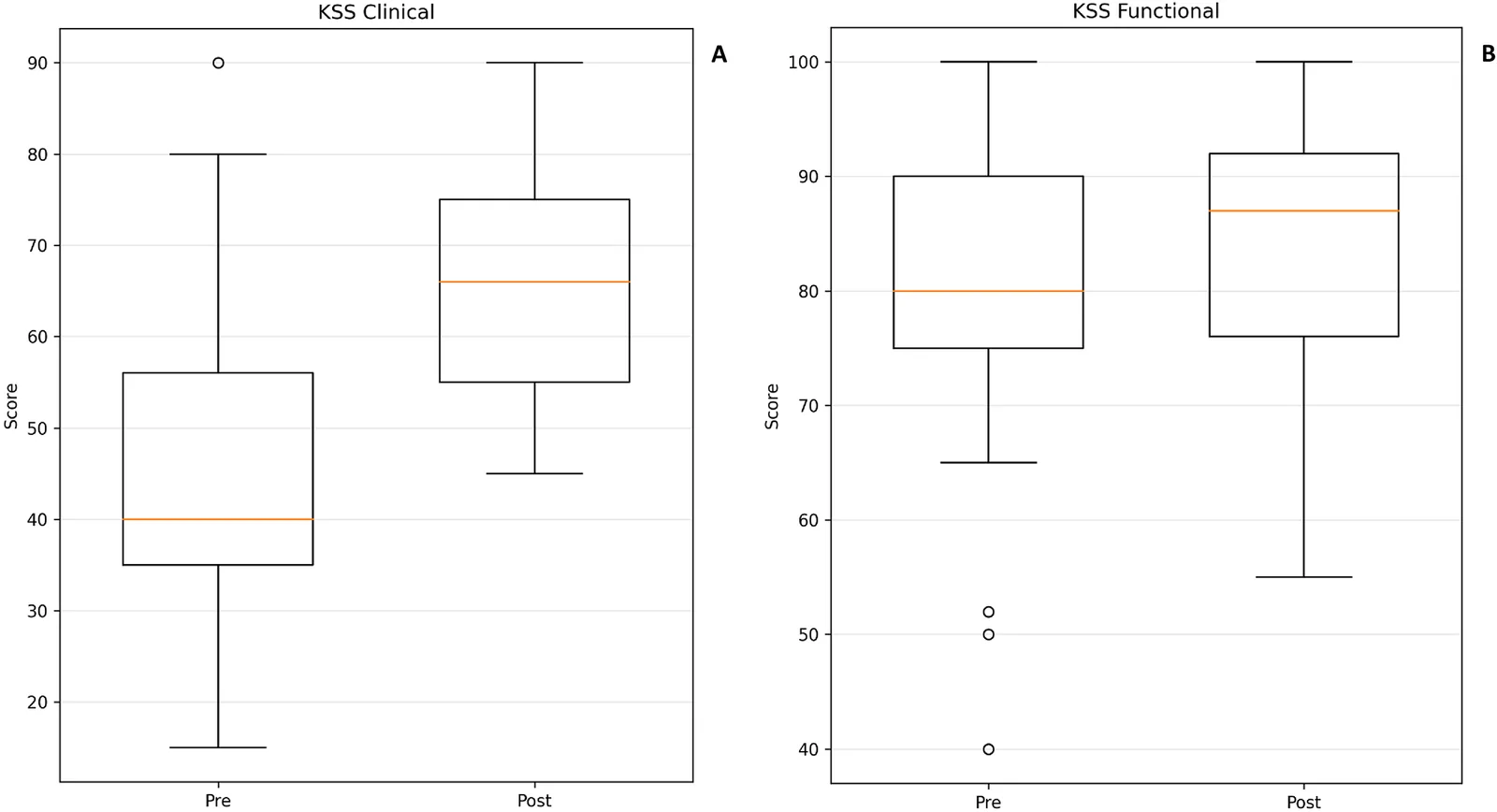
Preoperative and postoperative Knee Society Score (KSS) clinical (A) and functional (B) scores.

Postoperative satisfaction averaged 33.1 ± 6.6/40. ROM was overall preserved, with median value unchanged at 130°; mean ROM showed a slight decrease (133.0 ± 5.2°–128.9 ± 8.1°), corresponding to a mean change of −4.1° (95% CI, −7.2° to −1.1°), largely driven by a few outliers, while 94.3% of knees remained within ±10° of baseline, indicating no clinically relevant loss of motion.

### Return to sport

Among 30 patients, 23 (76.7%) were sports-active preoperatively and eligible for RTS assessment. At final follow-up, 18/23 resumed sports (RTS rate 78.3%), while 5 (21.7%) did not; relative to the overall cohort, postoperative sports participation was 18/30 (60.0%). Sports were categorized by impact profile as low-impact, high-impact, or mixed. In the preoperative sports-active subgroup (*n* = 23), activities were low-impact in 10 patients (43.5%), high-impact in 4 (17.4%), and mixed in 9 (39.1%). Among patients who returned to sport (*n* = 18), distribution was low-impact in 6 (33.3%), high-impact in 4 (22.2%), and mixed in 8 (44.4%). The proportion of high-impact participants was maintained among returners, while mixed-pattern participation increased.

In the overall cohort, median Tegner Activity Scale remained 5 from baseline to final follow-up (5 [IQR, 4–6] vs 5 [IQR, 3.5–6]), although a small but statistically significant paired difference was observed (Wilcoxon signed-rank test, *p* = 0.04).

### Radiographic outcomes

Preoperative mean baseline parameters were: HKA 179.6 ± 2.8°, trochlear sulcus angle 137.2 ± 8.3°, and congruence angle 28.4 ± 23.9°.

For paired preoperative-postoperative comparisons, patellar tilt decreased from 8.8 ± 6.7° to 6.4 ± 4.2°, mean change of −2.4 ± 9.3° (95%*CI* − 5.3° to 0.7°, without statistical significance (*p* = 0.139). Caton-Deschamps index changed from 1.1 ± 0.3 to 1.0 ± 0.1, mean change − 0.08 ± 0.32 (95%*CI* −0.19 to 0.03), without statistical significance (*p* = 0.23).

Patellar lateralization significantly improved, decreasing from 4.7 ± 3.8 to 0.8 ± 1.8mm (mean change −3.9 ± 3.5mm; absolute value 4.2 ± 3.1mm; 95% CI, 2.6–5.1 mm; *p* < 0.001).

ATOR index [12] was 0.12 ± 0.05 (Table 3).

## Discussion

The principal finding of this study is that RA-PFA for isolated PFOA was associated with encouraging early revision-free and reoperation-free survivorship; significant clinical improvement, including return to sport, preserved knee motion, improved patellofemoral tracking; and high concordance between planned and final component positions.

Modern PFA has regained interest with advances in implant design and improved patient selection [3]. However, outcomes remain highly dependent on indication and surgical precision [6]. As a compartment-specific procedure, PFA is particularly sensitive to errors in component size, rotation, coronal-sagittal positioning, and anterior offset, which may directly affect patellar tracking, overstuffing, pain, and implant survival [5, 6]. Contemporary functional concepts further emphasize patient-specific, three-dimensional component positioning integrating sagittal and rotational alignment, with the patellofemoral joint as a key determinant of knee function [13]. Robotic platforms enable preoperative planning and intraoperative assessment of implant positioning and alignment, improving precision and reproducibility.

Survivorship and safety profile observed in this cohort are consistent with the currently available literature on RA-PFA. Pacchiarotti et al. reported a revision-free implant survival of 94.4% at 52 months, with a conversion to TKA for tibiofemoral OA progression and no robot-related complications [14]. In the present series, Kaplan–Meier survivorship free from revision of any prosthetic component was 96.7% at 12 months and 91.6% at 24 and 36 months, while reoperation-free survivorship was 92.1% and 86.9%, respectively. Although comparisons are limited by differences in follow-up and cohort size, these findings align with recent reports of favorable short- to mid-term outcomes after RA-PFA [8, 9, 15]. Furthermore, the relatively short mean follow-up and decreasing numbers at risk beyond 24 months limit the precision of later Kaplan–Meier estimates.

Failure mechanisms remain relevant in the robotic era. Tibiofemoral OA progression, malposition-related instability, and patellar component failure are established causes of revision. [16] In this series, one revision was related to tibiofemoral OA progression, reinforcing the importance of strict indications and exclusion of femorotibial disease.

Beyond revision events, postoperative adverse events and pain-related symptoms occurred in a subset of patients; however, most were managed non-operatively and improved during follow-up, with only three knees requiring reoperation. Persistent postoperative pain represented the most frequent adverse event. Although patients underwent routine clinical and radiographic reassessment and identifiable causes were investigated when clinically suspected, no consistent mechanical, infectious, or implant-related cause emerged in most cases. These findings suggest that postoperative symptoms remain an important aspect of patient management after RA-PFA, while the overall complication profile remains in line with the encouraging early survivorship observed in this cohort. A key strength of this study is the evaluation of plan-to-execution accuracy. Deviations between planned and final implant positioning were minimal across all planes and proudness, with complete size concordance in evaluable cases. This supports the ability of the robotic workflow to reproduce the intended component position with minimal deviation between planned and intraoperatively recorded alignment parameters, which is particularly relevant in PFA given the sensitivity of patellofemoral biomechanics to small positional errors. Similar findings have been reported by Selvaratnam et al. [17]. Although these findings do not constitute independent radiographic validation of final implant position, they indicate high agreement between preoperative planning and the final intraoperatively recorded component position achieved within the robotic workflow. Compared with conventional PFA, which relies predominantly on manual instrumentation and surgeon-dependent intraoperative assessment, such reproducibility may represent a potential advantage of robotic assistance. However, whether this translates into superior clinical outcomes remains to be established. Clinically, RA-PFA was associated with substantial functional improvement, with significant gains in both clinical and functional KSS. Median ROM remained unchanged; the slight decrease in the mean value was driven by a few outliers rather than a global reduction, supporting maintenance of knee mobility. These findings are consistent with earlier reports showing meaningful postoperative improvement without ROM compromise [5, 10], which is particularly relevant in compartment-specific arthroplasty.

Return to sport was achieved in 78.3% of preoperatively active patients, consistent with reported rates of 64.7% to 91% in the literature [18]. Noyes et al. reported an 87% RTS rate in a younger RA-PFA cohort [7].

Whether robotic assistance may facilitate an earlier return to sport compared with conventional PFA remains unclear. Although improved component positioning and patellar tracking may theoretically support better postoperative recovery, available studies primarily report overall RTS rates rather than time to RTS, and direct comparative evidence remains lacking. The present study cannot determine whether robotic assistance enables an earlier return to sport or improves return-to-sport rates compared with conventional PFA. The statistically significant paired difference in Tegner score despite an unchanged median value likely reflects a small subgroup of patients with decreased postoperative activity levels, whereas most patients maintained their preoperative activity level. Therefore, the observed statistical significance should be interpreted in the context of the largely unchanged activity profile of the cohort.

Radiographic findings supported a favorable effect of RA-PFA on patellofemoral biomechanics. Preoperatively, this cohort showed a pattern of patellar maltracking, including increased patellar tilt and lateral displacement. Postoperatively, patellar lateralization improved significantly, whereas patellar tilt showed a favorable trend and patellar height remained unchanged, suggesting correction of patellar maltracking without patellar height alteration. These results are consistent with previous studies showing improved patellofemoral alignment after PFA [5, 7, 9, 10]. Present radiographic findings are consistent with the concept that robotic planning and execution may help optimize trochlear component positioning and thereby improve patellofemoral tracking, although the specific effect of robotic assistance cannot be determined without a control group.

Another relevant radiographic finding was the low postoperative ATOR value. Aweid et al. have linked higher ATOR values to anterior overstuffing and worse outcomes [12]. In this cohort, the mean ATOR (0.12 ± 0.05) suggests satisfactory restoration of anterior offset and low risk of overstuffing. Although ATOR should not be interpreted as an isolated determinant of outcome, it provides additional radiographic support for the technical adequacy of implantation in this series.

This study has several limitations. Its retrospective single-center design introduces potential selection and information bias. The relatively small sample size and short follow-up period limit the interpretation of survivorship outcomes and do not permit long-term conclusions. In the absence of a conventional PFA control group, it is not possible to determine whether the observed outcomes are attributable to robotic assistance itself or simply reflect the results of contemporary PFA performed in appropriately selected patients. The inclusion of five bilateral cases, analyzed as independent observations, may have introduced within-patient dependence and should be considered when interpreting the results. Finally, the fact that all procedures were performed in a high-volume tertiary center may limit the generalisability of these findings to other centers.

Overall, these findings suggest that CT-based RA-PFA is a promising option for carefully selected patients with isolated PFOA, providing encouraging early survivorship, functional improvement, preserved motion, improved patellofemoral tracking, and high agreement between planned and intraoperatively recorded component position. However, the specific effect of robotic assistance cannot be determined without a control group. Longer follow-up and comparative studies are required to determine whether these advantages translate into improved long-term outcomes.

## Conclusion

Image-based RA-PFA was associated with encouraging early survivorship, clinically relevant improvement, preserved knee motion, and improved patellofemoral tracking. However, postoperative symptoms and secondary procedures occurred in a subset of patients and should be considered when interpreting these findings. Longer-term multicentre comparative studies are required to determine whether these favorable early clinical, radiographic, and technical findings translate into superior long-term outcomes, and to clarify the specific role of robotic assistance in this technically demanding procedure.

## Abbreviations

ATOR: Anterior Trochlear Offset Ratio
BMI: Body Mass Index
CI: Confidence Interval
CRPS: Complex Regional Pain Syndrome
CT: Computed Tomography
HKA: Hip-Knee-Ankle
IQR: Interquartile Range
KSS: Knee Society Score
MPFL: Medial Patellofemoral Ligament
PFA: Patellofemoral Arthroplasty
PFOA: Patellofemoral Osteoarthritis
RA-PFA: Robotic-Assisted Patellofemoral Arthroplasty
RIO: Robotic Arm Interactive Orthopedic System
ROM: Range of Motion
RTS: Return to Sport
TKA: Total Knee Arthroplasty
TTO: Tibial Tubercle Osteotomy

## Data Availability

Data associated with this study are not publicly available due to ethical and privacy restrictions but may be available from the corresponding author upon reasonable request.
